# Action on elevated natriuretic peptide in primary care: a retrospective cohort study

**DOI:** 10.3399/BJGPO.2024.0017

**Published:** 2025-02-12

**Authors:** Cornelia JC Vermeer, Monika Hollander, Anne JM Stolk, Amy Groenewegen, Geert-Jan Geersing, Frans H Rutten, Huberta E Hart

**Affiliations:** 1 Department of General Practice & Nursing Science, Julius Centre for Health Sciences and Primary Care, University Medical Centre Utrecht, Utrecht University, Utrecht, The Netherlands; 2 Leidsche Rijn Julius Healthcare Centres, Utrecht, The Netherlands

**Keywords:** heart failure, general practice, natriuretic peptides, primary healthcare

## Abstract

**Background:**

Natriuretic peptides (NPs) are released by increased ventricular wall stress, most often caused by heart failure (HF). NP level measurement helps select patients clinically suspected of HF who need echocardiography. Yet, the diagnostic actions following NP testing in daily primary care are poorly studied.

**Aim:**

To assess the diagnostic actions taken by GPs in patients with an elevated NP level.

**Design & setting:**

Retrospective observational study in general practices in The Netherlands.

**Method:**

In patients with an elevated NP level between July 2017 and July 2022, diagnostic actions were collected during 3 months following NP testing. We compared patients with an elevated NP level referred for echocardiography with those not referred by univariable analyses.

**Results:**

Among 902 patients, 394 (43.7%) had an elevated NP level. Median age was 75.0 (interquartile range [IQR] 18.0) years; 68.8% were female. In total, 166 (42.1%) were referred for echocardiography and 114 (28.9%) underwent additional electrocardiogram (ECG) recording. In total, *n* = 30/166 (18.1%) referred patients were labelled HF by the cardiologist within 3 months after NP testing compared with *n* = 29/228 (12.7%) not referred. Referred patients were compared with those not referred and they were found to be younger (69.7 versus 74.1 years, *P*<0.001), were less often known to cardiologists (45.8% versus 62.3%, *P* = 0.002), and they had lower marginally elevated B-type natriuretic peptide (BNP) levels (35–50 pg/ml) (19.3% versus 36.6%, *P*<0.001).

**Conclusion:**

Three out of five patients with an elevated NP level are not referred for echocardiography by GPs. Barriers to refer patients were older age, a marginally elevated BNP value, and already being under supervision of a cardiologist.

## How this fits in

Early recognition of heart failure is crucial for the timely initiation of prognosis-improving treatment, which can alleviate symptoms, improve quality of life, slow disease progression, and ultimately reduce mortality. Guidelines recommend referring patients with elevated natriuretic peptide (NP) levels for echocardiography to confirm or exclude heart failure, regardless of the patient’s age or the degree of elevation. Three out of five patients with elevated NP values were (incorrectly) not referred for echocardiography by GPs. Older age, a marginally elevated brain natriuretic peptide (BNP) value, and already being under supervision of a cardiologist were related to non-referral.

## Introduction

Heart failure (HF) is a complex clinical syndrome mainly affecting older people. In high-income countries, the prevalence of known HF in the general population is around 2%, but when accounting for unrecognised cases, the calculated prevalence rises to 4.2%.^1,2^ In those aged ≥65 years, the prevalence of known HF is increased to 11.8%.^1,2^ Based on the findings of selective screening studies, it is calculated that in nearly half of the patients with HF, the condition remains unrecognised, often because symptoms and signs are misclassified, certainly at an early stage when patients are seen in primary care.^1–3^ Unfortunately, at the time of late diagnosis, detoriation has often already occurred owing to disease progression or acute cardiovascular events.

The GP plays an essential role in diagnosing HF, and the vast majority is detected in general practice. In patients with shortness of breath, reduced exercise tolerance, fatigue, and/or peripheral oedema, HF should be considered; however, these symptoms are non-specific.^4,5^ Notably, signs of fluid overload, for example, elevated jugular venous pressure, bilateral crackles with lung auscultation, and/or peripheral oedema, are often lacking in the early stages of HF, certainly in those with HF with preserved ejection fraction (HFpEF).^4,5^ Given the advancements in treatment options for HF, including for HFpEF, with the latest addition being sodium-glucose cotransporter 2 (SGLT2) inhibitors,^5–7^ enhancing early recognition and subsequent treatment of HF is pivotal to improve prognosis.

In 2005, the Dutch primary care guidelines on HF already recommended natriuretic peptide (NP) testing if GPs considered HF on a clinical basis.^8^ The revised 2010 and 2021 Dutch primary care HF guidelines, along with the 2012, 2016, and 2021 European Society of Cardiology (ESC) guidelines on HF, explicitly emphasised the importance of NP testing as well as electrocardiography (ECG) as the initial steps of HF diagnosis.^4,5,9–11^ Echocardiography should follow in those with NP values above the exclusionary cut-point (N-terminal pro-B-type natriuretic peptide [NT-proBNP] ≥125 pg/ml or B-type NP [BNP] ≥35 pg/ml) and/or an abnormal ECG.

Whether GPs follow these guideline recommendations regarding NP testing in daily primary care practice is poorly studied. The aim of this study was to assess what diagnostic actions GPs took in patients with an elevated NP level. Insight into the follow-up in patients with an elevated NP level could help to detect barriers and facilitators in the diagnostic work-up of HF in primary care.

## Method

### Design and setting

We performed a retrospective observational study using primary care data extracted from electronic health records (EHRs) of five Julius Health Centers in Leidsche Rijn (Leidsche Rijn Julius Gezondheidscentra; LRJG), a suburb of Utrecht, The Netherlands, including nearly 50 000 enlisted individuals. These general practices have access to laboratory testing including NP, ECG, and open-access echocardiography. In The Netherlands, all citizens are registered with a GP except those living in a nursing home, and the GP is gatekeeper for hospital specialist care. GPs use the International Classification of Primary Care (ICPC) coding to record diagnoses. They need to manually add ICPC codes to their electronic health record (EHR).

### Study population

The study population included all individuals registered at LRJG who (i) underwent NP testing between July 2017 and July 2022, (ii) were still enlisted in the practice at July 2022, and (iii) were not labelled with HF in the GP’s EHR before NP testing, defined as lacking the ICPC code K77 for HF.

### Data extraction

All data were collected in the summer of 2022. The level of NP and date of testing were registered. In case of multiple NP tests, the first measurement was considered for analysis. We used the exclusionary cut-off values for BNP (35 pg/ml) and NT-proBNP (125 pg/ml), as recommended by the European Society of Cardiology (ESC) and national HF guidelines.^4,5^ The medical records of patients who had NP measurements were systematically scrutinised for the following: (i) symptoms and signs suggestive of HF;^4^ (ii) subsequent diagnostic actions taken; (iii) a cardiologist’s diagnosis of HF; and (iv) a new ICPC code K77 for HF in the GP’s EHR within 3 months after NP testing.^10^

Shortness of breath, reduced exercise tolerance, fatigue, orthopnoea, nocturnal dyspnoea, nocturia, peripheral oedema, and pulmonary crackles were scored positive or negative based on registration in the GP’s EHR.

We assessed diagnostic actions taken within 3 months after NP testing and additionally recorded all ECGs performed 1 month before and 1 month after NP testing. An ECG was categorised as either normal or abnormal based on the interpretation of the physician who assessed the ECG. We further assessed referral for open-access echocardiography, and telephone contact with or referral to a cardiologist.

A cardiologist’s diagnosis of HF based on echocardiography was considered as true diagnosis of HF. Such a diagnosis could be extracted from the cardiologist’s letter that is routinely sent to the patient’s GP. In the Dutch healthcare setting, this approach is ‘standard of care’ for all patients referred to a hospital specialist or to an open-access echocardiography facility. The cardiologist’s letters are documented in the GP’s EHR and were assessed by one of the researchers.

Data on baseline characteristics (sex, age, body mass index [BMI], blood pressure, smoking status), and participation in integrated primary care disease management programmes for chronic obstructive pulmonary disease (COPD), type 2 diabetes (T2D), or cardiovascular risk management (CVRM) were retrieved from the GP’s EHR. The following ICPC codes were collected: anaemia (B80, B81, B82); angina pectoris (K74); prior myocardial infarction (K75); other ischaemic heart disease (K76); atrial fibrillation (K78); valvular heart disease (K83); hypertension (K85, K86, K87); COPD (R95); T2D (T90.02); dyslipidaemia (T93); and chronic kidney disease (CKD (IU99.01).

We also registered laboratory testing for estimated glomerular filtration rate (eGFR) when tested in the same blood sample or the test result most close within the past 5 years. An eGFR <60 ml/min/1.73m^2^ was considered reduced.^12^

### Data analyses

Descriptive statistics, including counts with corresponding percentages for dichotomised variables, and means with standard deviations or medians with interquartile ranges (IQR) for continuous variables, were computed to summarise baseline characteristics, comorbidities, symptoms and signs suggestive of HF, and for diagnostic actions taken. Differences between patients referred for open-access echocardiography or to a cardiologist and those not referred were compared using Pearson’s χ^2^ test or Fisher’s exact test for dichotomised variables and with independent samples t-test or Mann-Whitney U-test for continuous variables. A *P* value<0.05 was considered statistically significant. For data analysis R studio (version 2023.09.0) was used.

## Results

**Figure 1.**
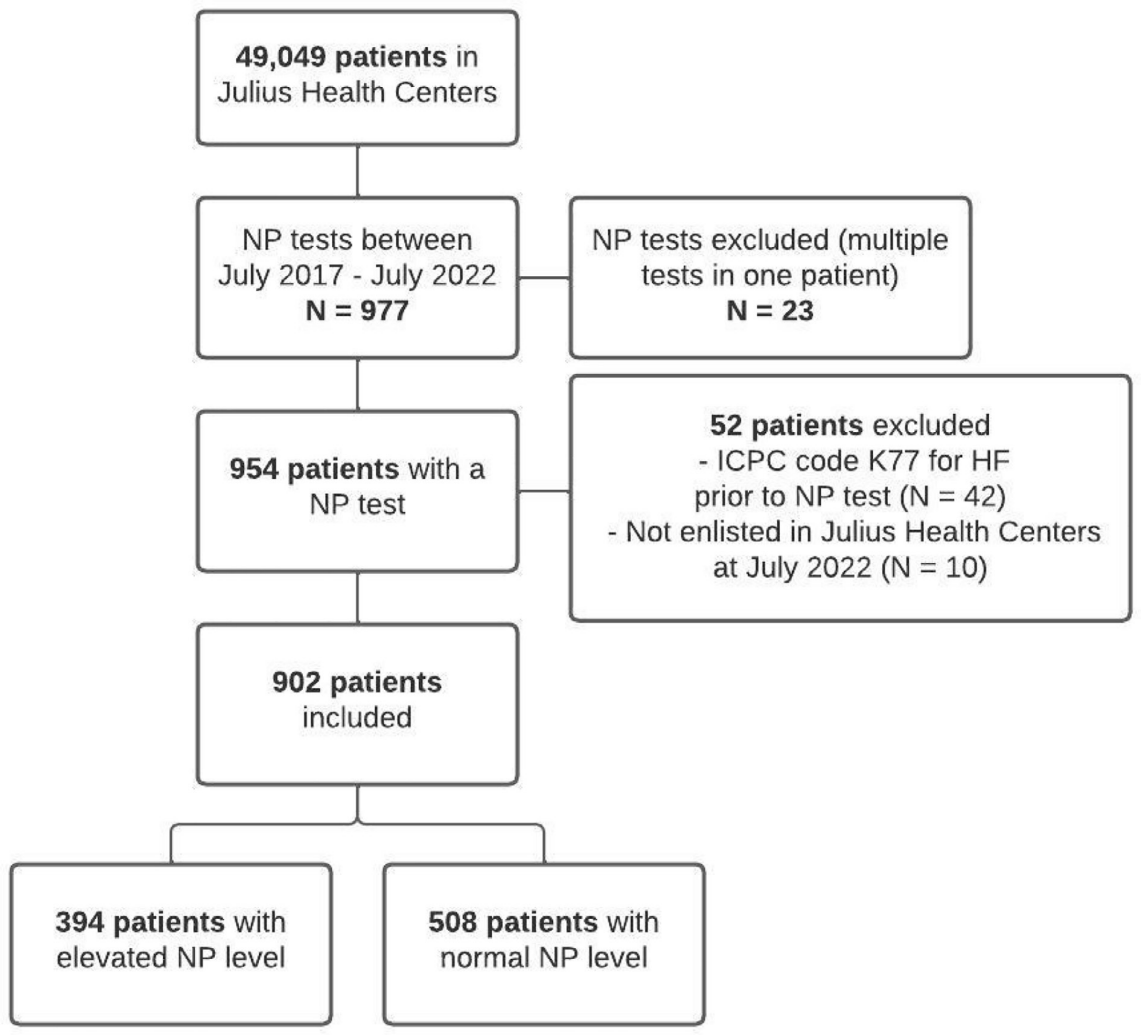
Flowchart of the study population. HF = heart failure. NP = natriuretic peptide

In total, 49 049 patients were registered in LRJG in 2017, and 902 (1.8%) patients underwent a NP measurement between July 2017 and July 2022 (Figure 1). In 94.6% patients BNP (median 30 pg/ml, IQR 147 pg/ml) was tested and in 5.4% NT-proBNP (median 86 pg/ml, IQR 125 pg/ml) was tested.

In 394 (43.7%) patients the NP level was above the exclusionary cut-point. The median age of these 394 patients was 75.0 (IQR 18.0) years, 68.8% were females, 56.9% had hypertension, and 65.0% participated in one or more of the integrated primary care disease management programmes (COPD, T2D, CVRM). The most commonly reported symptom was shortness of breath (50.0%), followed by peripheral oedema (40.1%) and reduced exercise tolerance and/or fatigue (37.8%). On physical examination, peripheral oedema was reported in 32.0%.

### Diagnostic actions

**Figure 2.**
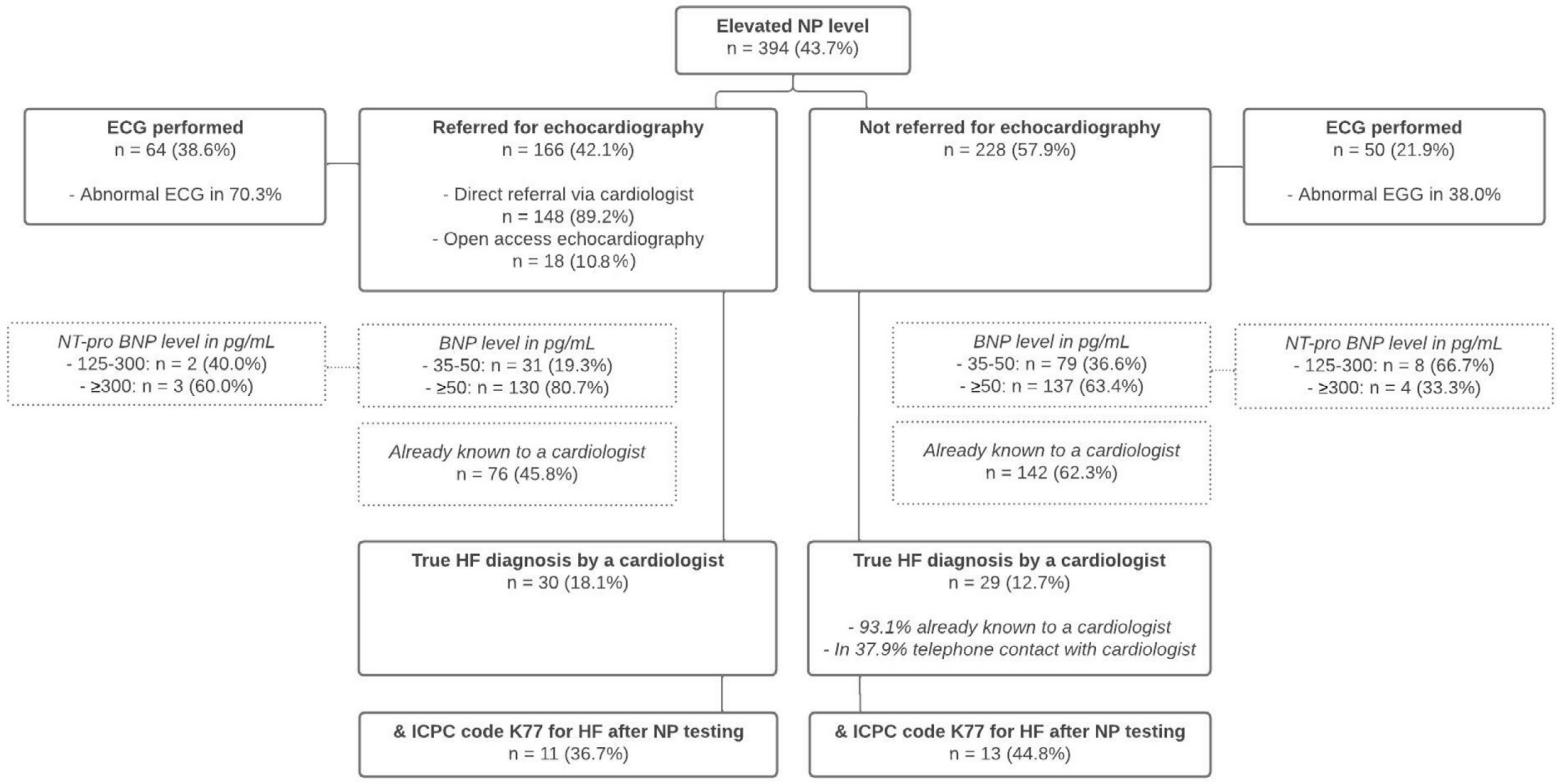
Overview of diagnostic actions taken by GPs in 394 patients with an elevated natriuretic peptide level. BNP = B-type natriuretic peptide. ECG = electrocardiogram. HF = heart failure. ICPC = International Classification of Primary Care. NP = natriuretic peptide

In *n* = 114/394 (28.9%) patients with an elevated NP level an ECG was performed, and the ECG was abnormal in 58.2%. GPs referred *n* = 166/394 (42.1%) patients for echocardiography; *n* = 18/166 (10.8%) to an open-access facility of which six were subsequently referred to a cardiologist, and *n* = 148/166 (89.2%) by direct referral to a cardiologist (Figure 2). In *n* = 120/148 (81.1%) patients the cardiologist made a (new) echocardiogram. In *n* = 60/394 (15.2%) patients with an elevated NP level there was telephone contact with a cardiologist about the elevated NP level.

### Referral for echocardiography

Patients referred for echocardiography were younger (69.7 years versus 74.1 years, *P*<0.001), less often had prior myocardial infarction (6.0% versus 14.9%, *P* = 0.009), and more often reported reduced exercise tolerance and/or fatigue (44.6% versus 32.9%, *P* = 0.024) compared with those not referred.

We observed no differences in other symptoms and signs suggestive of HF (Table 1). Patients referred for echocardiography also had more often an abnormal ECG (70.3% versus 38.0%, *P* = 0.002), and were less often known to a cardiologist before NP testing (45.8% versus 62.3%, *P* = 0.002), compared with those not referred. Patients with a slightly elevated BNP level (35–50 pg/ml) were less often referred to a cardiologist than those with levels≥50 pg/ml (19.3% versus 36.6%, *P*<0.001; Table 2).

**Table 1.** Patient characteristics of 394 individuals with an elevated natriuretic peptide level, subdivided in those referred and those not referred for echocardiography

	Referred *n* = **166**	Not referred *n* = **228**	*P* value
Median age in years (IQR)	73±21.8	78±17.3	<0.001
Median NT-proBNP in pg/ml (IQR)	400.0±348.0	219.0±193.8	0.104
Median BNP in pg/ml (IQR)	83.0±86.0	63.0±83.0	<0.001
Female	121 (72.9)	150 (65.8)	0.164
Mean BMI in kg/m^2^ (SD) (*n* = 320)	29.7±6.7	28.1±5.2	0.024
Mean SBP in mmHg (SD) (*n* = 385)	135.3±17.5	135.2±19.3	0.938
Mean DBP in mmHg (SD) (*n* = 385)	80.3±12.1	78.6±10.9	0.154
**Smoking (** ***n*** **= 307),** ***n*** **(%)**			
Never Past Current	68 (52.7) 50 (38.8) 11 (8.5)	90 (50.6) 76 (42.7) 12 (6.7)	0.798 0.566 0.714
**Comorbidities *n* (%)**			
Anaemia	24 (14.5)	35 (15.4)	0.919
Angina pectoris	22 (13.3)	27 (11.8)	0.791
Prior myocardial infarction	10 (6.0)	34 (14.9)	0.009
PCI or CABG	9 (5.4)	11 (4.8)	0.972
Atrial fibrillation	34 (20.5)	51 (22.4)	0.745
Valvular heart disease	16 (9.6)	21 (9.2)	1.00
Hypertension	95 (57.2)	129 (56.6)	0.980
COPD	14 (8.4)	33 (14.5)	0.095
Type 2 diabetes	37 (22.3)	53 (23.2)	0.919
Dyslipidaemia	30 (18.1)	39 (17.1)	0.908
Chronic kidney disease	36 (21.7)	63 (27.6)	0.220
**Participation in integrated PC disease management programme *n* (%**)			
Total	103 (62.0)	153 (67.1)	0.351
COPD programme	7 (4.2)	14 (6.1)	0.540
Type 2 diabetes programme	32 (19.3)	50 (21.9)	0.607
CVRM programme	69 (41.6)	101 (44.3)	0.662
**Blood test values *n* (%**)			
eGFR<60 ml/min/1.73 m^2^ (*n* = 388)	32 (19.9)	60 (26.4)	0.169
**HF suggestive symptoms**			
Shortness of breath	92 (55.4)	105 (46.1)	0.083
Reduced exercise tolerance and/or fatigue	74 (44.6)	75 (32.9)	0.024
Orthopnoea and/or nocturnal dyspnoea	19 (11.4)	14 (6.1)	0.090
Nocturia	21 (12.7)	28 (12.3)	1.00
Peripheral oedema	68 (41.0)	90 (39.5)	0.846
**HF suggestive signs**			
Pulmonary crackles	24 (14.5)	26 (11.4)	0.456
Peripheral oedema	53 (31.9)	73 (32.0)	1.00

Data presented as mean±standard deviation (SD), median±interquartile range (IQR), or absolute count (%). BMI = body mass index. CABG = coronary artery bypass graft surgery. COPD = chronic obstructive pulmonary disease. CVRM = cardiovascular risk management. DBP = diastolic blood pressure. eGFR = estimated glomerular filtration rate. HF = heart failure. NT-proBNP = N-terminal pro-B-type natriuretic peptide. PC = primary care. PCI = percutaneous coronary intervention. SBP = systolic blood pressure.

**Table 2.** Potential factors affecting the GP's decision on referral for echocardiography in patients with an elevated natriuretic peptide level

*n* (%)	Referred *n* = **166**	Not referred *n* = **228**	*P* value
BNP slightly elevated (35–50 pg/ml)	31/161 (19.3)	79/216 (36.6)	<0.001
NT-proBNP slightly elevated (125–300 pg/ml)	2/5 (40.0)	8/12 (66.7)	0.593
Abnormal ECG	45 (70.3)	19 (38.0)	0.002
Known to cardiologist before NP testing	76 (45.8)	142 (62.3)	0.002
Telephone contact with cardiologist after NP testing	31 (18.7)	29 (12.7)	0.138

Data presented as absolute count (%). ECG = electrocardiogram. NT-proBNP = N-terminal pro-B-type natriuretic peptide. NP = natriuretic peptide

### HF diagnosis

Among patients with an elevated NP level, HF was diagnosed by a cardiologist in *n* = 59/394 (15.0%) patients; 30 out of 166 (18.1%) patients were referred for echocardiography and 29 out of 228 (12.7%) patients were not referred for echocardiography within 3 months after NP testing. In *n* = 27/29 (93.1%) patients diagnosed with HF, but not referred for echocardiography after NP testing, this diagnosis was already known and based on echocardiography. These patients were already under the care of the cardiologist. In two patients, the diagnosis was only based on the clinical assessment and elevated NP levels (287 pg/ml and 280 pg/ml, respectively) (Figure 2). In 11 of these 29 (37.9%) patients who were not referred for echocardiography, the GP contacted the cardiologist by phone about what to do with the elevated NP results, and it was decided not to perform echocardiography by shared decision.

### Registration of HF diagnosis in the GP’s EHR

Out of the 59 patients with a true HF diagnosis by a cardiologist, in 10 (16.9%) patients (three referred and seven not referred), HF was already diagnosed by a cardiologist. However, this diagnosis was not registered with the ICPC code K77 for HF in the GP’s EHR at the time of NP level measurement.

Within 3 months after NP testing, the ICPC code K77 for HF was registered in the GP’s EHR in 49 of 394 (12.4%) patients with an elevated NP level. Of the patients with a true HF diagnosis by a cardiologist, this occurred in 24 out of 59 (40.7%); in 11 (36.7%) of those referred for echocardiography, and in 13 (44.8%) of those not referred for echocardiography. See also Figure 2.

## Discussion

### Summary

NP testing is not often performed in routine general practice, just 1.8% of the general population underwent such testing over a 5-year period. In 43.7% of these patients the NP level was above the ESC HF guidelines’ exclusionary cut-point. Among those with an elevated NP level, nearly half of the patients were referred for further echocardiography testing (4.6% open access, 37.6% directly via the cardiologist), as recommended by HF guidelines.

HF was considered diagnosed in 59 (15%) patients with an elevated NP level; in 30 (50.1%) patients referred for echocardiography, in 27 patients not referred but already known with a cardiologist’s diagnosis of HF, and in two patients based on clinical assessment and elevated NP levels.

### Strengths and limitations

This study provides insight into the frequency of NP testing in routine general practice, and what GPs decide to do if NP values are elevated.

A limitation is that we did not investigate potential barriers for non-adherence to the recommendation to perform echocardiography in patients with an NP level above the exclusionary cut-point. To address this adequately, a qualitative study would be necessary. Second, we did not account for the decision-making process; we do not know whether the referral was based on shared decision making with the patient. Third, GPs have to manually label the ICPC code based on the diagnosis mentioned in the specialist letter, and even for an important chronic progressive disease such as HF, this is not always done, or not within due time. Consequently, 10 patients who underwent NP testing and lacked an ICPC code K77 for HF were already known with HF by the cardiologist and thus misclassified in the GP’s EHR.^13^ This finding, that specialist diagnoses are not always registered as such in the GP’s EHR, is in line with routine care and previously reported in a Dutch observational study on HF in general practice.^14^

### Comparison with existing literature

Measurement of NP has a significant diagnostic contributing effect in primary care patients suspected of HF.^15–17^ In a cross-sectional diagnostic study reporting on how often GPs performed additional investigations in patients suspected of new-onset HF, almost 30% had HF, and NT-proBNP showed to be the most powerful diagnostic test.^15^ NPs are also used to guide treatment decisions. In the two recent landmark randomised controlled trials (RCTs) conducted in patients with HF with mid-range ejection fraction (HFmEF) and HFpEF (HF and a left ventricular ejection fraction [LVEF] >40%; EMPEROR-Preserved and DELIVER), the inclusion criterion was NT-proBNP levels >300 pg/ml.^18,19^ In these patients, SGLT2 inhibitors significantly improved the combined endpoint of cardiovascular death and HF hospitalisations. Finally, NPs can be used for risk-stratified management. In the STOP-HF RCT, intensification of cardiovascular risk management in patients with BNP levels >50 pg/ml had a significant beneficial effect in that it decreased the risk for developing HF, and also major adverse cardiovascular events (MACE) and emergency cardiovascular hospital admissions within 1–3 years.^20,21^ These studies underscore the importance of NP measurements and adhering to clinical HF guidelines with referral of patients with NP levels above the exclusionary cut-point for open-access echocardiography or to a cardiologist to confirm or refute HF.^4,5^

Despite that open-access echocardiography has been available for two decades, only a minority (4.6%) of patients with an elevated NP level in our study were referred to this facility, thus missing the opportunity to decrease referrals to cardiologists and increase the GPs’ capacity to manage HF themselves.^21^

Important when interpreting our results is the finding that 62.3% of patients with an elevated NP level not referred for echocardiography were under the care of a cardiologist, and in some of these, it seems that the GP was unaware that the patient had HF, given that the ICPC code K77 for HF was lacking in the GP’s EHR. This underscores the need for improved communication about patients’ clinical status between cardiologist and GP, but also for adequate ICPC labelling in the GP’s EHR of specialist diagnoses.

Finally, concerning our finding that patients with a marginally increased NP value were less likely to be referred for echocardiography, it is noteworthy that other conditions, such as higher age, atrial fibrillation, and renal dysfunction, could have withheld referral, knowing that these conditions can also result in especially marginally elevated NP levels.^10^ The median age (75.0 years, IQR 18.0) of the patients with an elevated NP level was rather high, and this might have caused GPs to refrain from referral for echocardiography.^22^ Although BNP levels <35 pg/ml or NT-proBNP levels <125 pg/ml are useful to exclude HF, the specificity and positive predictive value are very low at these exclusionary cut-points, and cannot support a diagnosis of HF.

### Implications for practice

There seems to be ample room for improvement in the follow-up of NP testing in primary care. Education of GPs and practice nurses may help improve the (early) diagnosis of HF. Certainty about the diagnosis and identification of the type of HF significantly contributes to improved management of these patients.^5–7^ In addition to enhancing knowledge of HF diagnosis and when to measure NPs, general practice could also benefit from financial incentives for NP measurement and referral for echocardiography when NP levels are elevated in adults with shortness of breath and fatigue. Financial incentives can help mitigate barriers such as time constraints for GPs. Moreover, widespread availability of open-access echocardiography and optimising cooperative care with cardiologists can further facilitate the diagnostic process in patients suspected of HF by the GP. For example, the reporting of echocardiography could be improved by using terminology that is more familiar and accessible for GPs, and it should include a clear recommendation on what to do for a specific patient. Finally, uniformity in exclusionary cut-points and units across laboratories facilitates accurate interpretation of NP levels and supports effective clinical decision making of GPs in the diagnostic work-up of patients suspected of HF.

In conclusion, three out of five patients with an elevated NP level are not referred for echocardiography by GPs. Barriers to refer patients were older age, a marginally elevated BNP value, and being already under supervision of a cardiologist.

There is ample room for improvement in diagnostic testing of patients with elevated NP values, but also in the ICPC labelling in the GP’s EHR of those with established HF.

## Data

The dataset relied on in this article is available from the corresponding author on reasonable request.
